# Utilization of CBCT to improve the delivery accuracy of Gamma Knife radiosurgery with G‐frame

**DOI:** 10.1002/acm2.13332

**Published:** 2021-06-30

**Authors:** Lindsey Claps, Damien Mathew, Kathryn Dusenbery, Margaret Reynolds, Yoichi Watanabe

**Affiliations:** ^1^ Department of Medical Physics MSKCC New York Ctiy NY USA; ^2^ Department of Radiation Oncology University of Minnesota Minneapolis MN USA

**Keywords:** CBCT, coordinate definition accuracy, Gamma Knife ICON, G‐frame

## Abstract

**Purpose:**

To quantify the G‐frame based stereotactic coordinate definition accuracy of Leksell coordinate G‐frame‐based Gamma Knife radiosurgery (GKRS) by the on‐board cone‐beam CT (CBCT) and establish remedial action rules to minimize the delivery errors.

**Methods:**

We analyzed the data of 108 patients (a total of 201 tumors) treated by GKRS with G‐frame for head fixation. After co‐registering the CBCT images and plan reference images, the Leksell GammaPlan (LGP) treatment planning system provided the amount of geometric translation and rotation required to minimize the position difference between the plan and treatment. The software also calculated maximum displacement, which characterizes the position shift more clearly. We studied how much these predicted dosimetric quantities changed if the treatment was delivered without correcting the patient's position.

**Results:**

The maximum displacement of the patient position obtained from the co‐registration of CBCT and plan reference images was 0.81 ± 0.38 mm (0.24–2.03 mm). The target coverage decreased by 3.3 ± 7.0% on average (−48.5% to +35.7%). The decrease of the target coverage, however, became smaller as the target volume increased. In particular, if the volume was greater than 2 cm^3^, the %change in target coverage was always less than −5%.

**Conclusions:**

The position differences reported by the registration module of LGP were within the accuracy limit of image registration for most clinical cases, but the errors could be larger in some cases. Therefore, we propose the following decision process. We do not advise position adjustment for G‐frame based GKRS if the maximum displacement is less than 1 mm. When this limit is exceeded, however, another criterion should be applied to the decision making by considering the tumor size (or the treatment volume) together with the acceptable change of the tumor coverage.

## 1 INTRODUCTION

Stereotactic radiosurgery (SRS) techniques are routinely used to treat brain tumors with excellent local control. Among those, Leksell Gamma Knife (LGK) (Elekta AB, Stockholm, Sweden) has been considered one of the most accurate systems for SRS for the past thirty years.^1^ The recent introduction of newer models, i.e., Perfexion and ICON, enables fractionated SRS. The ICON model utilizes on‐board cone‐beam CT (CBCT), an infrared (IR) light‐based motion tracking system (High Definition Motion Management, HDMM),^2^ and a thermoplastic mask to immobilize the patient's head, obviating the need for an invasive stereotactic frame such as the Leksell coordinate frame G (or G‐frame). LGK‐ICON users can still utilize the G‐frame‐based SRS delivery for single fraction treatments, especially for very small tumors because it is considered the most accurate for coordinate definition of the stereotactic space for planning and treatment.

Older versions of the Leksell GammaPlan (LGP) treatment planning system required users to mount a skull‐scaling instrument, or “bubble,” onto the G‐frame to estimate the position of the scalp relative to *x*, *y*, and *z* treatment coordinates and define the shape of the skull. Bubble measurements taken after the frame was first placed were compared to measurements prior to treatment. A significant variation in these measurements suggested that the frame may have moved prompting an investigation into the frame integrity which could potentially require remounting of the G‐frame and obtaining a new imaging scan. Newer versions of LGP, i.e., Version 11 and higher, however, allow for the use of CT or MR images to define the shape of the skull, foregoing the need of bubble measurement, and thereby losing an essential check of G‐frame integrity.

With the introduction of the ICON model, users have a more accurate method for confirming the quality of frame fixation prior to starting treatment. CBCT images are acquired before the start of treatment and co‐registered with the plan reference images. Ideally, there should be no geometric translation or rotation if the frame has not moved and if the pins are not loose; a variance might indicate there has been movement of the head between the time of frame placement and the start of treatment.

In order to determine thresholds above which an interventional action is required before proceeding with treatment delivery, we collected the shift and rotation angles and the impact on the DVH data for patients treated with the ICON system. After analyzing these data, we determined a variance level that suggested movement in the frame and warranted further investigation prior to delivering treatment.

## 2 METHODS AND MATERIALS

### 2.1 Clinical cases

We collected co‐registration data for 108 patients undergoing GKRS with the G‐frame head fixation with a total of 201 tumors (the average of 1.9 tumors per patient). With the patient in the head frame, a 1.5‐T Magnetom MRI with a FLASH pulse sequence (Siemens Healthineers) was performed with the fiducial box in place. The pixel size was 0.5 ×0.5 mm and slice thickness was 1 mm. The study was approved by the Institutional Review Board (IRB) of our institution for data analysis.

### 2.2 CBCT and plan delivery quality

Before treatment delivery, patients were scanned by the on‐board CBCT system. The acquired images were co‐registered with the planning MR image sets, using the image registration module of LGP. After registration, the software displays the translational shifts in mm for three orthogonal directions *x* (left to right), *y* (anterior to posterior), and *z* (superior to inferior), and three rotational angles in degrees around three axes (roll, pitch, and yaw). Those geometrical translations and rotations are required to match the plan CT/MR images with CBCT. Additionally displayed is the maximum displacement, which is the maximum displacement of the shot positions used in the plan. The maximum displacement considers not only the translational shifts but also the rotation angles. It is noted that the software cannot apply the shift and angle data for readjustment of the patient's head when the treatment is using the G‐frame for head fixation.

Once the co‐registration is approved after inspection of the co‐registered image sets, the software displays the values of plan quality parameters for the final treatment plan position, as well as the treatment plan before the position adjustment. The latter values indicate the dosimetric qualities of the treatment delivery if the position is not adjusted based on the registration information. The quality parameters of all targets/tumors are calculated. The six quality parameters are: the minimum, maximum, and mean doses, the target coverage, the Paddick conformity index (PCI), and the gradient index (GI). To evaluate the extent of changes in these parameters, we calculated the relative percentage differences of the minimum dose and the mean dose. The changes of the target coverage and PCI were calculated by taking the difference of two values in percentage, denoted as %change hereafter.

### 2.3 Data analysis

We analyzed the translation and rotation data. There were 19 quantities available for this study. These quantities can be grouped into four categories, as follows.

1. Coordinate definition: the mean and maximum displacement of the coordinate system defined by using the fiducial markers in the MR indicator box (min_disp and max_disp).
2. Plan parameters: the treatment and tumor volumes (txt_vol and tumor_vol), the target coverage in % (coverage).
3. Co‐registration parameters: three translational shifts and three rotation angles displayed on the registration window of LGP after the CBCT is co‐registered with the plan CT/MRI, (*x*, *y*, *z*, pitch, yaw, and roll), the maximum displacement (max_shift), and the vector length calculated from three translational shifts (vector_shift).
4. Treatment quality changes: the change in minimum, maximum, and mean doses of the target (min_dose, max_dose, and mean_dose), the target coverage (Coverage), the Paddick Conformation Index, PCI, (Padix_Index), and the GI (Grad_Index).

We did data analysis of these quantities by taking the statistical correlations among the co‐registration parameters and the treatment quality parameters and by generating a linear regression model. For the statistical analysis, we used the corrplot and lm modules in the R‐package.^3^

## 3 RESULTS

**Figure 1:**
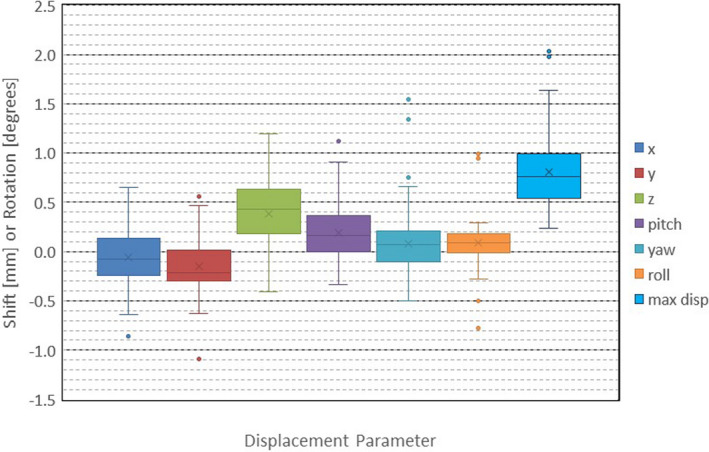
Box–Whisker plot of translation shift and rotation data of 108 patients

The data of the geometrical translation and rotation are summarized in Figure 1 for the 108 patients (201 tumors). The Box–Whisker plot shows the range of changes in seven parameters, *x*, *y*, and *z* for the translational shift in mm, pitch, yaw, and roll angles for the rotation in degrees, and the maximum displacement in mm after the CBCT image was co‐registered with the plan MRI image. In the figure, “*x*” indicates the mean, and the short horizontal line in the box indicates the median. The box shows the range of 25% and 75% quartiles. The horizontal lines on the ends of whiskers show the upper and lower extremes. Solid circles indicate the outliers. The median values of the shift parameters were smaller than 0.5 mm, with the most significant change of the shift in the *z*‐direction. It is understood that the translational shift in the *z*‐direction was large because the slice thickness of the planning MRI image was 1 mm while the pixel size on the axial plane was 0.5 ×0.5 mm. Furthermore, note that the voxel size of the CBCT image was 0.4 mm in all three orthogonal directions. The mean of the maximum displacement was 0.81 ± 0.38 mm (range: 0.24–2.03 mm).

**Figure 2:**
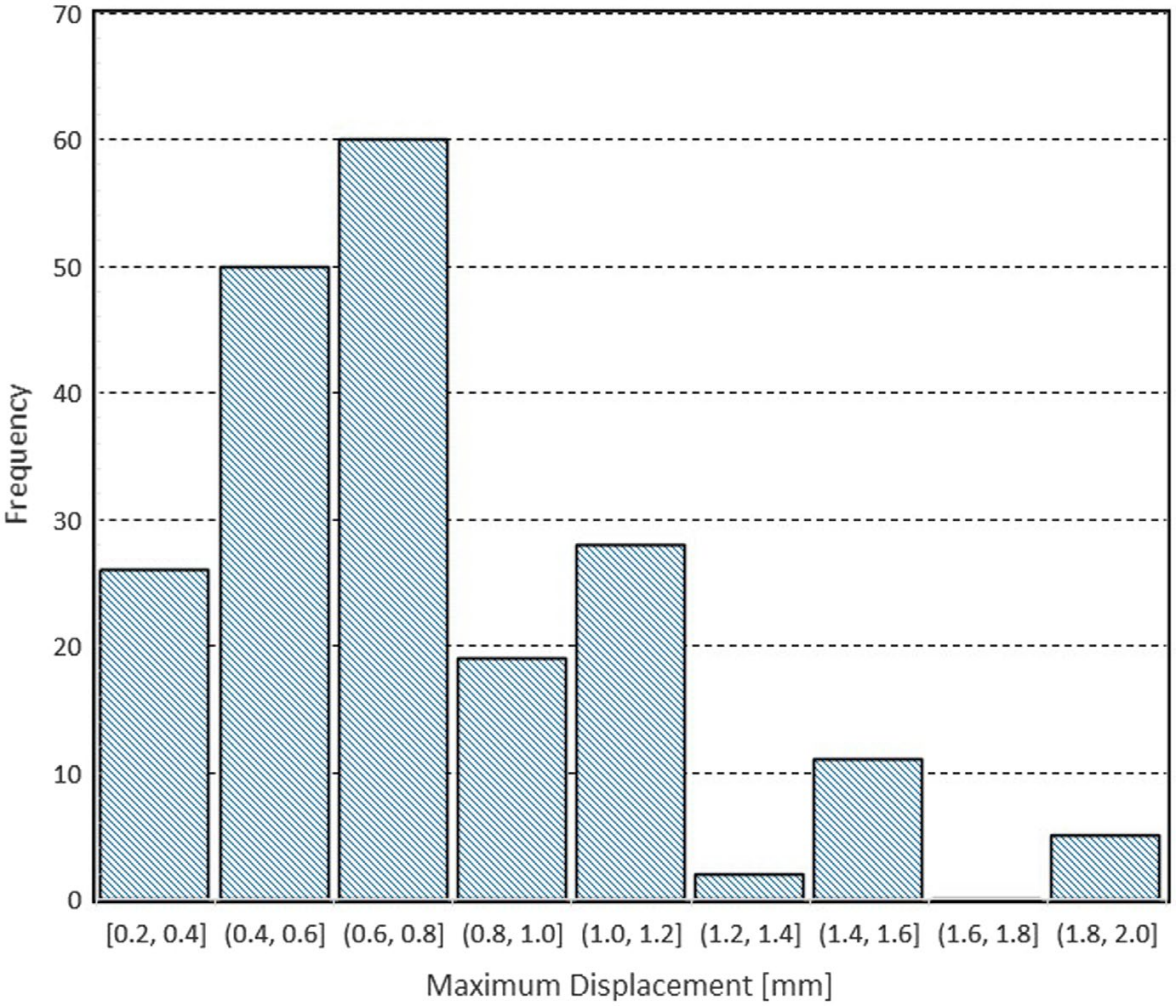
Histogram of the number of tumors vs. the maximum displacement

The histogram of Figure 2 shows the number of tumors for a varying amount of the maximum displacement required for the optimal registration of the plan reference images and the CBCT images. The largest number of tumors, 60, had the maximum displacement in the range between 0.6 and 0.8 mm. There was no case with larger than 2.1‐mm maximum displacement.

**Figure 3:**
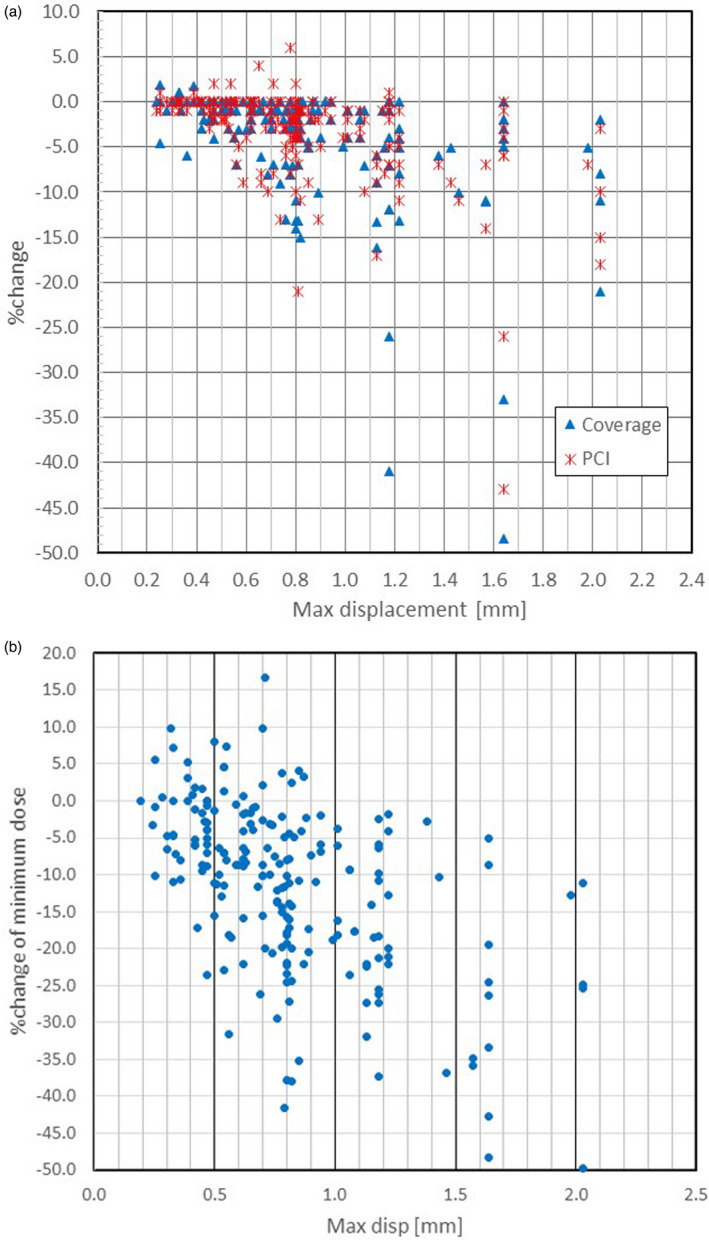
%change in (a) target coverage and Paddick conformity index (PCI), (b) minimum target dose vs. maximum‐displacement for 201 tumors

Figure 3a shows the percent change (%change) of both the target coverage (Coverage) and the PCI as a function of the maximum displacement. We noticed the target coverage correlated with the maximum displacement. Coverage and PCI decreased with increasing maximum displacement. This meant that the delivered treatment quality worsens in comparison to the planned treatment as the maximum displacement increases. Although the data points are scattered widely, we can notice that %change was smaller than −5% if the maximum displacement was smaller than 0.4 mm, except one case of Coverage. The current clinical data show that the target coverage decreased by 3.3 ± 7.0% on average (range: −48.5% to +35.8%) if the position correction was not made. In one instance, coverage increased by 35.8% when displacing shots. This occurred for a trigeminal neuralgia treatment, in which the target coverage of the plan was small (and not the parameter that is typically maximized for this treatment), and the shift of the image improved the coverage considerably.

Figure 3b is similar to Figure 3a, but it is for the %change in the minimum target dose. It shows the same trend as the target coverage and PCI, but the magnitude of changes was larger than the former. The mean change was −11.5 ± 11.5% on average (range: −49.8% to +16.7%). Even the maximum displacement of 0.47 mm resulted in 23.6% decrease of the minimum dose in one case.

**Figure 4:**
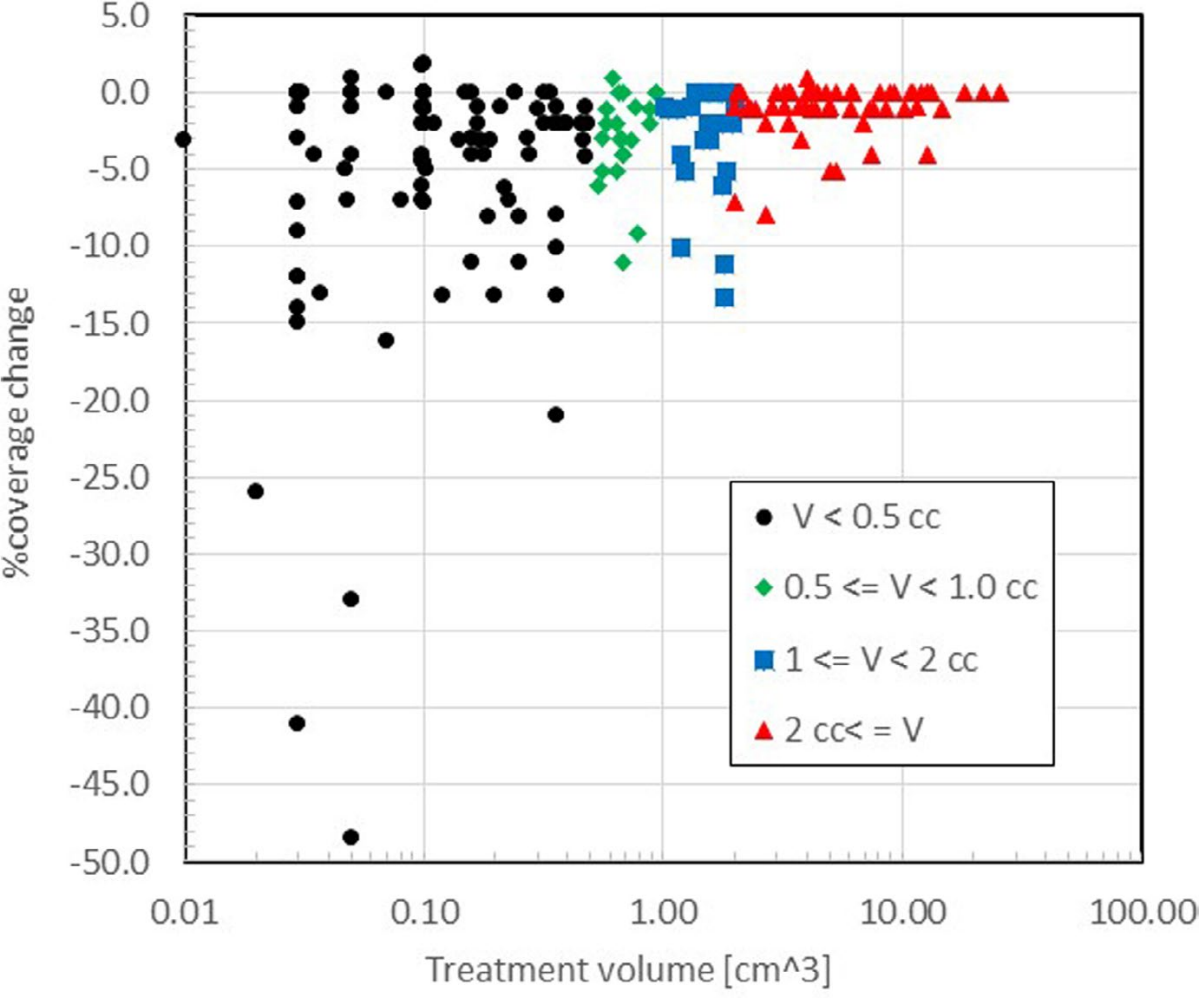
%change in target coverage and PCI vs. treatment volume of 201 tumors

Figure 4 shows the %change in the target coverage as the function of the treatment volume (*V*). For this plot, the tumors were divided into four groups according to the treatment volume; Group 1: *V* < 0.5 cm^3^, 2: 0.5 cm^3^ ≤ *V* < 1.0 cm^3^, 3: 1.0 cm^3^ ≤ *V* < 2.0 cm^3^, 4: 2 cm^3^ ≤ *V*. The figure clearly shows that the larger the volume, the smaller the %change in target coverage. In particular, if the volume was greater than 2 cm^3^, the %change in target coverage was always less than −5%, except two cases (2 out of 58, or 3.4%). On the other hand, for small tumors (volume smaller than 0.5 cm^3^), the % change in target coverage ranged from 1.9% to −48.5%.

Combining the results presented in Figures 3 and 4, we can come to the following two conclusions when we accept only cases with less than a 5% change in the target coverage. First, regardless of the value of maximum displacement, almost all cases with the treatment volume greater than 2 cm^3^ can meet the 5% criterion. Secondly, for a treatment volume smaller than 2 cm^3^, only cases with the maximum displacement smaller than 0.4 mm can result in less than 5% change of the target coverage.

**Figure 5:**
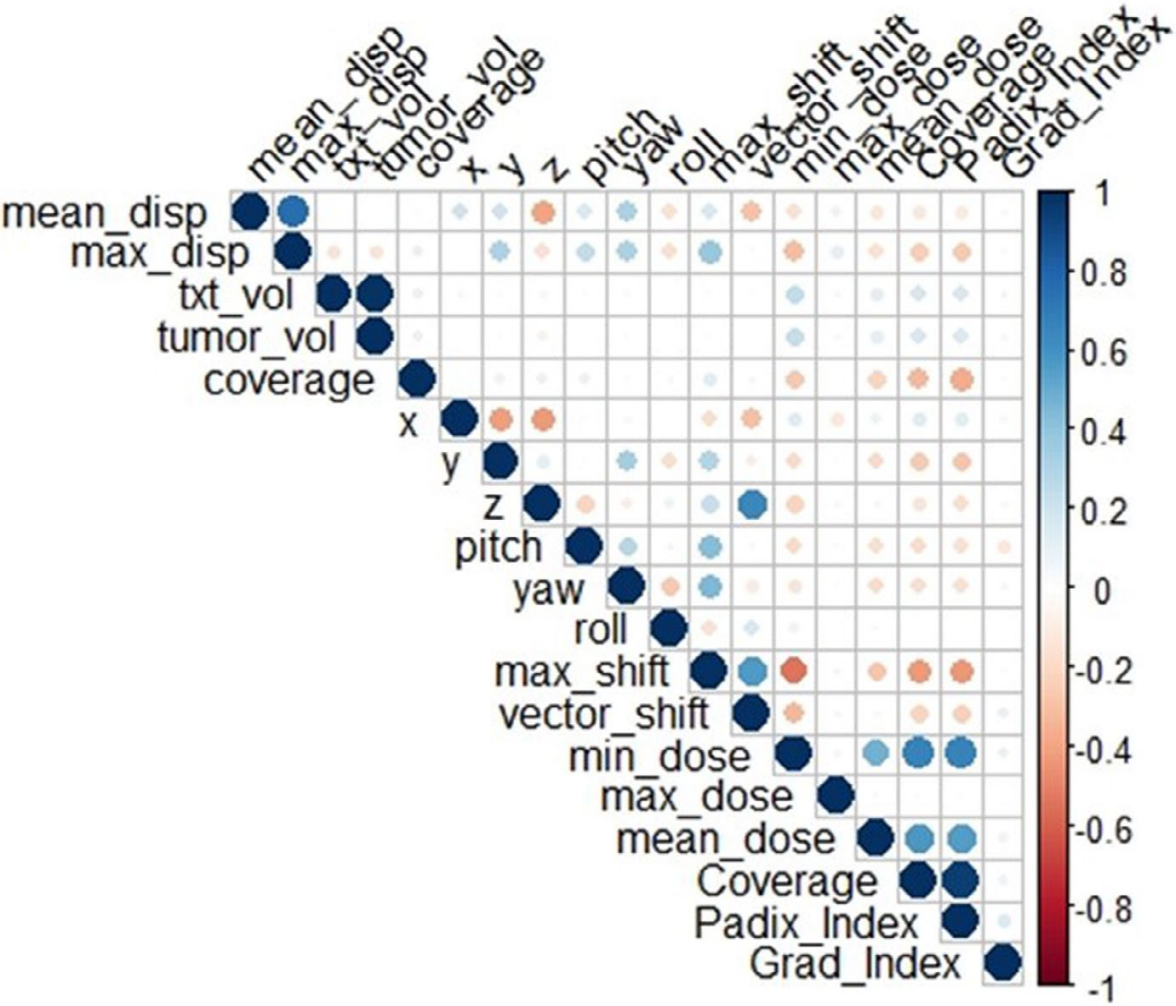
Correlation plot among 19 parameters for 201 tumors. The size and brightness of filled circles in a cell of the triangular lattice indicate the level of correlation between two quantities at the top and left sides of the lattice. The higher the correlation, the larger and brighter the circle. The color of blue and red indicate positive and negative correlation, respectively

Figure 5 shows the statistical correlation between the 19 parameters described in Section 2. From the graph, we can observe the following:

- The mean_disp and max_disp were highly correlated with each other in a positive sense, but did not have a strong influence on other parameters.
- The *z*‐shift had a significant impact on the vector_shift.
- There was a high positive correlation between Coverage and Padix_Index.
- The max_shift was negatively and strongly correlated with min‐dose, Coverage, and Padix_Index.

The observations made in Figure 5 as well as the results shown in Figure 4 suggested that the change in the target coverage was a function of the treatment volume and the maximum displacement. A linear regression model for predicting the change in the target coverage as a function of treatment volume (txt_vol) and maximum displacement (max_shift) was developed. The resulting regression equation was

(1) $$Change\;in\;Coverage=A*Treatment\;Volume\;\left[{\mathrm{cm}}^{3}\right]+B*Maximum\;displacement\;\left[mm\right]+C$$

where the coefficients *A*, *B*, and *C* with the *p* values were given by

*A* = 0.003164 ± 0.001113 (*p* = 0.00494),

*B* = −0.079024 ± 0.011570 (*p* < 1.02e‐10),

*C* = 0.023740 ± 0.010574 (*p* = 0.02586).

The two coefficients *A* and *B* of those two variables were estimated with high statistical significance or very small *p* value, as shown above. The adjusted *R* ^2^ value of 0.207 indicates the model is not perfect but adequate for the estimation of the Change in Coverage.

## 4 DISCUSSION

The geometrical mismatch between the CBCT images and the plan reference MRI of patients with G‐frame was found by co‐registering those two image sets. The translations and rotations required to correct the mismatch were large. Without the correction, the dose delivery quality degraded so much that, for some treatments, the tumor coverage was not sufficient.

We aimed to establish a sound rule for determining when the MRI to CBCT fusion resulted in unacceptable calculated deviation that suggested a problem with the frame stability or integrity. Let us assume that the amount of the “shift” or displacement due to the combined effects of the translation and the rotation can be quantified by a single quantity denoted by ∆. This shift can then be represented by a function of four types of errors: the inherent error due to the imperfect definition of the 3D coordinate system using the MR/CT localizer with MR/CT images, δ_coord_; the displacement of the G‐frame relative to the head from the time the frame was placed, to the time of CBCT image acquisition, δ_frame_; the error in the image registration, δ_fusion_; and the inherent inaccuracy of the CBCT imaging system, δ_CBCT_. By assuming these errors are independent, the quantity ∆ can be expressed by

(2) $$\Delta ={\left(\delta_{\mathrm{coord}}^{2}+\delta_{\mathrm{frame}}^{2}+\delta_{\mathrm{fusion}}^{2}+\delta_{\mathrm{CBCT}}^{2}\right)}^{1/2}$$

The accuracy of the 3D coordinate definition by using the localizer box with G‐frame is influenced by the geometrical quality of the images containing visible fiducial markers. Occasionally, the error can be induced by G‐frame distortion due to excessive torque on the frame and posts during G‐frame placement.^4^ A comprehensive study undertaken by Mack et al. showed δ_coord_ of 0.48 mm.^5^ Since their measurements included the dose delivery error, the true geometric error could be smaller. Here, we assumed 0.5 mm for δ_coord_ as a conservative estimate. The frame‐related displacement can occur by the slippage of the G‐frame due to improper location of screws on the skull,^6^ or movement of the G‐frame due to screws that are too‐loose on the skull. Without these rare incidences, the displacement δ_frame_ is 0.6 mm or smaller.^7^, ^8^ It is noteworthy that the magnitude of the frame displacement during the treatment process may depend on the neurosurgeons. Our preliminary data indicated a statistically significant difference in δ_frame_ among neurosurgeons, depending on their experience level. Errors due to image co‐registration depend on the type of images, i.e., CT, CBCT, or MR. Furthermore, it is affected by imaging parameters and protocols, even for the same imaging modality. Errors published in the literature vary from 0.3 to 1.5 mm.^9^, ^10^, ^11^, ^12^ A recent paper for the ICON‐CBCT system states that errors are 0.5 ± 0.2 mm for CBCT‐CT fusion and 0.8 ± 0.3 mm for CBCT‐MRI fusion.^13^ Here, we used 0.8 mm for δ_fusion_. The geometric accuracy of CBCT imaging system is very high and δ_CBCT_ is estimated to be 0.2 mm or smaller.^14^ Note that our daily CBCT precision test data taken during the study period showed that the error ranged from 0.05 to 0.24 mm with the mean of 0.12 mm. When conservative estimates of these values are used, Equation (2) becomes:

(3) $$\Delta ={\left(0.5^{2}+0.6^{2}+0.8^{2}+0.2^{2}\right)}^{1/2}=1.14\;\mathrm{mm}$$

From the above analysis, it is reasonable to conclude that the average maximum displacement is about 1.0 mm for the co‐registration of the plan reference image and the CBCT image under the usual circumstance of a framed patient. Therefore, let us set the tolerance to 1 mm for the maximum displacement. Then, the data shown in Figure 3 suggest that 50 out of 201 tumors (or 24.9%), 17 out of 108 (or 15.7%) patients, need a reevaluation of frame placement quality before treatment.

To quantify the magnitude of errors expected when there is no slippage or displacement of the G‐frame during the entire GKRS procedure, we used a spherical phantom filled with polymer gel. The phantom was placed in the G‐frame using four pairs of post and screws. The phantom was first scanned with CT and MRI. The phantom was then placed in the ICON unit for CBCT. By taking CBCT images and doing co‐registration of images, we measured the amount of displacement required for the best match of the image sets on LGP. The results showed that the means of all six displacement parameters were within ±0.5 mm for CT. However, when CBCT was co‐registered with MRI, the rotational errors were larger than 1 mm. Although such large errors were most likely caused by the near‐spherical geometry of the phantom, which made the co‐registration algorithm find the unique registration solution difficult, the errors were large. So, further quantification study is needed to make a definite conclusion. Despite this shortcoming, from the experiment, we may conclude that the cumulative error ∆ is less than 1 mm if there is no frame slippage from the experiment. On the other hand, by setting δ_frame_ to zero in Equation (2), we obtained 0.96 mm for this experiment.

Before we can set a tolerance value of the maximum displacement, we need to examine the effects of displacement on the dose delivery quality. The required plan quality or its change without a proper position adjustment of the G‐framed patient strongly influences the tolerance value. Let's assume that we can accept less than a 5% change in the dose delivery quality, such as the target coverage and PCI. Then, Figure 3 suggests that the allowable maximum displacement is about 0.4 mm. However, there are only 19 tumors (9.5%) or 15 patients (13.9%), which met the 0.4‐mm threshold of the maximum displacement. Hence, such a rule is not practical, and we need a more robust decision criterion for the re‐planning.

**Figure 6:**
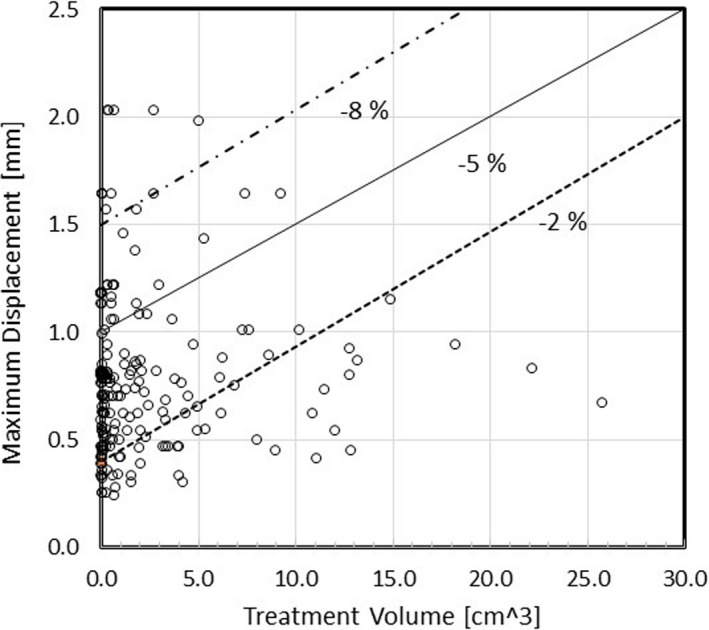
Iso‐tolerance lines in %change of target coverage (−2%, −5%, and −8%) as a function of the tumor treatment volume and maximum‐displacement. The locations of 201 tumors are indicated by empty circles

As seen in Figure 5, the target coverage, PCI, and the minimum dose were very sensitive to the translation and rotation errors; therefore, these can be used as the quantifier of dose delivery quality. Further, we showed that the change in the target coverage strongly depended on the treatment volume and the maximum displacement, and a linear regression equation of Equation (1) can present the relationship. Using this equation, we can determine the maximum displacement tolerance for a given treatment volume for various changes in the target coverage, as shown in Figure 6. Three oblique lines correspond to −2%, −5%, and −8% change in the target coverage. For example, if the volume is very small or less than 0.5 cm^3^, the maximum displacement of even 1 mm is not acceptable to achieve less than a 5% decrease in the target coverage. But this value increases to 1.5 mm if the target volume is 10 cm^3^. Figure 6 also shows all 201 tumors as empty circles. Tumors below the −5% line meet the requirement of 5% or less change in the target coverage. There are 24 tumors (11.9%) above the −5% line. The G‐frame placement of the patients with those tumors must be reexamined before continuing treatment. Note that these decision criteria lead to a lower number of problematic tumors (24 tumors) than just considering the maximum displacement tolerance of 1 mm (50 tumors).

Based on the data taken at our institution and applying a rough estimation of the overall uncertainty, we found that about one in 10 patients needs further intervention, including re‐planning before the treatment. To decide if re‐planning is necessary, we need to check four items. Firstly, we should confirm the accuracy of the image registration by examining the relative displacement of easily identifiable landmarks on the original images (MR or CT) and CBCT. Potentially useful landmarks are the sharp edges of bony structure like the skull and the small circular dots of blood vessels. The outlines of the skull are rather easy to delineate on CBCT, CT, and MRI. The blood vessels are visible often as small black or white circles on T1‐ and T2‐weighted MRI. Secondly, we should confirm if the G‐frame is securely attached to the adapter of the Icon treatment couch. Thirdly, we should check if the G‐frame is firmly attached to the head. It is often easy to visually confirm an unsteady G‐frame by shaking it. Lastly, we should inspect the CBCT for the position of the screws or take the patient for a CT scan to confirm the screw placement on the skull.^6^

Re‐planning is time‐consuming and undesirable for both patients and providers. There are several approaches to minimize the need to repeat the procedure consisting of the reframing, rescanning, and re‐planning. The frame displacement errors caused by the frame deformation can be easily avoided by measuring the torque of screws during the frame placement.^4^ Careful handling of the patient before the start of treatment is needed. Expecting the known uncertainty discussed in this article, we might add a margin, such as 1 mm, for the treatment of a G‐framed patient to reduce the problematic warnings. Alternatively, we can stop the use of the MR/CT indicator box for coordinate definition by using CBCT even with G‐framed patients as proposed by Duggar et al.^15^ Yet, another solution is to enable the LGP to adjust the head position using the geometrical translation and rotation data obtained by co‐registration of CBCT and plan reference image sets with the G‐framed patient as done with all masked patients.

## 5 CONCLUSIONS

In this study, we proposed the tolerance value for the maximum displacement, above which the continuation of treatment is discouraged unless adequate corrective actions are taken. The position differences estimated by co‐registration of plan MRI with CBCT are within the accuracy limit of image registration, 1 mm, for most cases, i.e., about 75% of tumors. Hence, we do not advise position adjustment for G‐frame‐based GKRS using the position correction suggested by the LGP, as long as the maximum‐displacement is within 1 mm. When the indicated maximum‐displacement is larger than 1 mm, however, another criterion should be applied to the decision making by considering the tumor size (or the treatment volume) together with the acceptable change of the tumor coverage.

## CONFLICT OF INTEREST

None.

## AUTHOR CONTRIBUTIONS

Linsey Claps: analysis and interpretation of data and writing and revising the paper. Damien Mathew: analysis and interpretation of data and writing and revising the paper. Kathryn Dusenbery: treatment planning and delivery and writing and revising the paper. Margaret Reynods: treatment planning and delivery and writing and revising the paper. Yoichi Watanabe: conception and design of the study and analysis and interpretation of data and writing and revising the paper and final approval of the manuscript.

## Data Availability

The data that support the findings of this study are available from the corresponding author upon reasonable request.
